# Convalescent plasma therapy in COVID-19 patients: a non-randomized case-control study with concurrent control

**DOI:** 10.1590/1414-431X2022e12235

**Published:** 2022-09-12

**Authors:** P. Cacilhas, E. Caberlon, L. Angoleri, K. Fassina, R.N. Ribeiro, L.C. Pinto

**Affiliations:** 1 Hospital Nossa Senhora da Conceição, Porto Alegre, RS, Brasil

**Keywords:** Convalescent plasma, COVID-19, Non-randomized trial

## Abstract

Convalescent plasma therapy has shown controversial results in coronavirus disease-19 (COVID-19) patients. We performed a non-randomized case-control study with contemporaneous controls in a hospital in southern Brazil. Patients were selected for treatment with convalescent plasma by medical decision and compared with patients who did not receive plasma and were hospitalized due to COVID-19 at the same time. The outcomes of interest were intensive care unit (ICU) admission and in-hospital death. Patients that received convalescent plasma had lower in-hospital mortality than patients that did not receive plasma (relative risk (RR) 0.48; 95% confidence interval (CI) 0.29 to 0.79) and these results were consistent after changing the subset of control patients. There were no differences regarding ICU admission between groups (RR=0.80; 95%CI: 0.47 to 1.35). In this study, patients that received convalescent plasma for COVID-19 had lower in-hospital mortality, but this finding requires further confirmation given the retrospective nature of the study.

## Introduction

Since late 2019, coronavirus disease (COVID-19) has been a global public health concern, and finding an effective treatment is still a cause for great debate and research. Few strategies have demonstrated benefit, while several have failed (1-3). One of the strategies that looked particularly interesting from the beginning was the use of convalescent plasma, and this and other treatments have shown controversial results (4-6). Most of the trials were conducted with hospitalized patients and failed to demonstrate the benefit of this therapy (4). Reasons for failure included time to plasma infusion and plasma antibodies titers (6,7). Nonetheless, a recent study from Argentina has shown that ambulatory patients with COVID-19 who received convalescent plasma up to 3 days after diagnosis of infection had less development of severe respiratory disease (6).

Given the emergency for treatment and the controversial results, we evaluated the efficacy of convalescent plasma therapy in hospitalized patients with COVID-19 in a public setting in southern Brazil through a case-control study with concurrent control.

## Material and Methods

Physicians of the hospital had convalescent plasma available for prescription, with minimal antibody titer of 1:80 and could prescribe it for patients that fulfilled at least one of the following criteria: respiratory rate ≥30; peripheral oxygen saturation <93%; or partial pressure of oxygen (PaO_2_)/fraction of inhaled oxygen (FiO_2_) ratio ≤300 mmHg. Patients with previous allergic reaction to blood derivatives, patients with end-of-life resolution, and patients with severe organ dysfunction were excluded from the study. Patients that failed to give informed consent were also excluded. The decision to prescribe plasma or not was taken by the assistant physician. The researchers had no influence on patient care during hospitalization.

Patients that gave consent and had at least one of the criteria received a single dose of convalescent plasma intravenously. These patients were matched in a 1:2 proportion with control patients. Control patients were also patients that were admitted in the hospital due to COVID-19 and who did not receive convalescent plasma by physician decision. In order to perform statistical analysis, these patients were matched to the plasma group according to their Charlson Comorbidity Index (CCI) (8). Outcomes of interest were admission to the intensive care unit (ICU) and in-hospital mortality. This study was approved by the Ethics Board of the hospital (CAEE 36249820.8.0000.5530).

Statistical analysis was performed in R software (version 4.0.3; R Core Team). Variables with normal distribution are reported as means±SD, while variables with non-normal distribution are reported as median and interquartile range (IQR). Categorical variables are reported as percentages. We used Student's *t*-test and Mann-Whitney test to compare continuous variables and Fisher’s exact test to compare proportions. An estimate of the relative risk and 95% confidence interval was also reported.

## Results

Patients assigned to the study were hospitalized between October 2020 and March 2021. All patients were unvaccinated for COVID-19 at the time of inclusion in this trial. In this period, twelve patients received convalescent plasma; the mean age was 57.8 years and 5 patients were female (41.7%). The control group consisted of 24 patients matched for CCI, with a mean age of 56.1 years, of which 13 patients were female (56.5%). Mean CCI in both groups was 1.5. The main characteristics of the patients are described in Table 1.

**Table 1:** Characteristics and outcomes of patients included in the study according to convalescent plasma therapy.

	Convalescent plasma therapy		P
	Yes (n=12)	No (n=24)	
Female, n (%)	5 (41.7)	13 (56.5)	0.48
Age, mean ± SD	57.8 ±11.4	56.1 ± 11.4	0.83
Hemoglobin, mean ± SD	12.7 ± 2.9	10.0 ± 3.8	0.04
Leukocytes, mean ± SD	6176 ± 3256	7794 ± 4377	0.26
D-dimers, median (IQR)	733 (629-1070)	1038 (683-1429)	0.40
C-reactive protein, median (IQR)	114 (50-185)	126 (75-178)	0.83
Lactate, median (IQR)	1.1 (0.9-1.6)	1.3 (1-1.9)	0.62
Creatinine, median (IQR)	0.95 (0.75-1.07)	0.82 (0.60-1.21)	0.48
Type 2 diabetes, n (%)	4 (33)	14 (60.9)	0.16
Hypertension, n (%)	7 (58.3)	17 (73.9)	0.45
Ischemic cardiac disease, n (%)	1 (8.3)	8 (34.8)	0.12
COPD, n (%)	3 (25)	1 (4.3)	0.21
Active cancer, n (%)	0 (0)	2 (8.7)	0.01
Obesity, n (%)	3 (25)	15 (65.2)	0.03
Treatments during COVID-19			
Glucocorticoids, n (%)	12 (100)	22 (95.7)	1
Antibiotics, n (%)	10 (83.3)	22 (95.7)	0.26
Outcomes			
ICU admission, n (%)	6 (50)	15 (65.2)	0.47
In-hospital death, n (%)	1 (8.3)	14 (60.9)	0.0003

Student's *t*-test, Mann-Whitney test, or Fisher exact test was used. SD: standard deviation; IQR: interquartile range; COPD: chronic obstructive pulmonary disease.

There was no difference in ICU admission between the plasma and control groups, with a relative risk (RR) of 0.80 (95% confidence interval (95%CI) of 0.47 to 1.35). However, in-hospital mortality was lower in patients that had convalescent plasma administered (RR=0.48; 95%CI: 0.29 to 0.79) (Table 1). As the plasma group had a borderline lower prevalence of obesity and lower prevalence of active cancer, we performed logistic regression to assess if the lower mortality in this group could be related to these characteristics, but results were similar after adjustment. Moreover, in order to confirm the results, patients that received plasma were matched with two different subsets of patients and the results remained statistically significant, with lower in-hospital death in the plasma group. There were no adverse events due to plasma infusion.

## Discussion

These results are somehow different from what has been published so far in hospitalized COVID-19 patients and convalescent plasma; none of the randomized trials to date has shown a benefit for plasma in terms of mortality (4,5,7). There are some differences among the studies and limitations of the current study that must be highlighted: the first is the non-randomized study design, which is subject to all inherent bias of such a study, mostly selection bias; to overcome this issue, we repeated the analysis with three different subsets of random patients and the results remained the same.

Moreover, the benefits shown by Libster et al. (6) were attributed to the early administration of plasma and high antibody titers; therefore, we also analyzed the antibody titer of plasma administered. Some patients received plasma with a titer of 1:80. In addition, as this study was conducted in a hospital setting, the mean days from symptoms to plasma infusion was 12 (except for two patients that had in-hospital positivity for SARS COV-2 infection and received plasma on the second day of symptoms), so this also did not explain our results. Another difference between our study and the others was the age of the participants: in our study, the mean age was 57 years, while patients in other studies had a mean age between 70-76 years (4,6). The last and perhaps most important difference was the higher mortality in our sample, 41% compared to 3.7 to 19% (4-7) in other studies. This difference could be related to the greater power of the current analysis. Moreover, the only randomized study that found some kind of benefit of plasma transfusion in COVID-19 was ambulatorial. The patients in that study were from Argentina, which has similarities to the population in southern Brazil. Another non-randomized study recently demonstrated a mortality benefit of plasma infusion, but the population consisted mainly of immunocompromised individuals and most patients had hematological cancer, which cannot be compared with the population of our study (9).

Some shortcomings of the current study must be addressed. Although not statistically significant, the number of patients with obesity and active cancer was unbalanced in the groups, which may have benefited the intervention arm. Because of the limited number of patients, when trying to balance these characteristics, other characteristics such as type 2 diabetes, COPD, and age of participants became unbalanced. Therefore, we decided to balance the comorbidities of the groups according to the CCI, which is a commonly used scale to analyze patients’ comorbidities. Three analyses were conducted with three different control groups and the intervention remained a protective factor (new control group paired for CCI: RR for death=0.16; 95%CI: 0.04 to 0.60; new control group unpaired for CCI: RR for death=0.04; 95%CI: 0.02 to 0.07). Another limitation that might have interfered with the results is the non-randomized nature of the study. The decision whether or not to prescribe convalescent plasma is in the hands of the assistant physician, which may introduce biases and differences in patient care.

### Conclusion

In this study, convalescent plasma therapy showed benefits in mortality in hospitalized patients due to COVID-19. Whether the observed mortality benefits are related to the population itself or due to selection bias could be a matter of debate and should be better clarified in a randomized trial.
